# Thrombotic Microangiopathy as an Emerging Complication of Viral Vector–Based Gene Therapy

**DOI:** 10.1016/j.ekir.2024.04.024

**Published:** 2024-04-15

**Authors:** Nora Schwotzer, Carine El Sissy, Isabelle Desguerre, Véronique Frémeaux-Bacchi, Laurent Servais, Fadi Fakhouri

**Affiliations:** 1 Service of Nephrology and Hypertension, Department of Medicine, Lausanne University Hospital and University of Lausanne, Lausanne, Switzerland; 2 Department of Immunology, Assistance Publique-Hôpitaux de Paris, Hôpital Européen Georges Pompidou, Paris, France; 3 Paris University, Paris, France; 4 Paediatric Neurology Department, Necker Hospital, APHP Centre, Université Paris Cité, Paris, France; 5 MDUK Oxford Neuromuscular Center and NIHR Oxford Biomedical Research Center, University of Oxford, Oxford, UK; 6 Neuromuscular Center, Department of Pediatrics, University of Liege and University Hospital of Liege, Belgium

**Keywords:** complement, gene therapy, immune response, thrombotic microangiopathy

## Abstract

Gene therapy has brought tremendous hope for patients with severe life-threatening monogenic diseases. Although studies have shown the efficacy of gene therapy, serious adverse events have also emerged, including thrombotic microangiopathy (TMA) following viral vector–based gene therapy. In this review, we briefly summarize the concept of gene therapy, and the immune response triggered by viral vectors. We also discuss the incidence, presentation, and potential underlying mechanisms, including complement activation, of gene therapy-associated TMA. Further studies are needed to better define the pathogenesis of this severe complication of gene therapy, and the optimal measures to prevent it.

Gene therapy has emerged as an innovative therapeutic tool for inheritable and noninheritable diseases. The basic concept of gene therapy is to deliver, via viral or nonviral vectors, genetic material to target cells, in order to correct or supplement defective genes, and thus improve or prevent diseases.^1^^,^^2^ Genetic material can be delivered as naked DNA or more efficiently in viral vectors that are able to transduce target cells.

Gene therapy has raised tremendous hopes for the treatment of severe disorders but carries the risk of potential serious complications.^3^ One of the emerging gene therapy–related complications of interest for nephrologists is TMA. In this mini-review, we first summarize the following: (i) the basics of viral vector–based gene therapy and viral vectors transduction of target cells, and (ii) the host immune response to viral vectors, which may lead to cellular injury, in particular endothelial cells. In the second part, we discuss the presentation and potential mechanisms of TMA associated with gene therapy.

### Viral Vector–Based Gene Therapy

In viral vector–based gene therapy, the viral genome is nearly entirely replaced with a therapeutic gene cassette. The virus is thus stripped of its replicative and pathogenic capacities. Gene transfer can be achieved *ex vivo* through the genetic engineering of harvested target cells that are infused back into the patient or *in vivo* through the direct delivery of the gene therapy product that will hopefully target specific cells. *In vivo* delivery can be local, loco-regional, or systemic, which triggers different potential safety issues.

In most cases, gene therapy is used to deliver a missing gene,^4^ or an abbreviated version of it^5^ in diseases caused by deficiencies in specific genes. More rarely, gene therapy aims to inhibit overexpressed genes and blunt the toxicity of the resulting misfolded proteins, the prototypic disorder being Huntington disease and the misfolded neurotoxic mutant huntingtin.^6^ Gene therapy may also aim to deliver RNA to specifically interfere with posttranscription, as in Duchenne Muscular Dystrophy.^7^ Gene therapy can also be used to deliver protective or corrective products, such as angiogenic vascular endothelial growth factor in arteriopathies or antiangiogenic molecules in wet age related macular degeneration.^8^

Gene therapy is usually delivered as a single high dose infusion (2 × 10^11^ to 2 × 10^14^ vector genomes per kilogram [vgs/kg]) of viral vectors. The dose is calculated based on body surface or weight and older children with higher body mass receive higher doses of viral vectors. For therapy targeting the central nervous system and delivered through lumbar puncture or intrathecal injection, a fixed dose of vector is generally used (∼1 × 10^14^ vgs)

### Viral Vectors Transduction of Target Cells

Several engineered replication-deficient viral vectors are used for gene therapy: retroviral and lentiviral vectors mainly for *ex vivo* gene therapy and adenovirus vectors and adeno-associated virus (AAV) vectors mainly for *in vivo* gene therapy. The choice of a viral vector depends on several parameters, including the spectrum of transduced cells and tissues, the packaging capacity, the risk of viral genome integration and subsequent insertional mutagenesis, and the propensity to induce host immune responses.

### AAV in Gene Therapy

AAV are currently the preferred vectors for *in vivo* gene therapy in clinical practice because they achieve efficient and sustained transfection of a wide variety of cells (due to the diversity of capsids variants), while eliciting a minimal host inflammatory reaction (as compared to adenoviral vectors, for example); the latter being a limitation for effective cell transduction. AAV are nonenveloped single stranded DNA parvoviruses,^9^ with a small icosahedral capsid (∼25 nm). Their genome (∼5 kb) is flanked by inverted terminal repeats, that are crucial for long lasting cell transduction and vector production. AAV require a helper virus (such as herpes simplex or adenovirus) for replication, and do not exhibit pathogenicity in humans.

More than 12 natural AAV serotypes and more than 100 variants have been identified,^10^ and despite sharing 60% to 99% identity, they display distinct cell tropism and transduction efficiency. It is estimated that more than 80% of adult humans have encountered AAV and 30% to 60% of them have neutralizing antibodies.^11^ However, immunoprevalence in early infancy is much lower (13%–17%).^12^^,^^13^ Several AAV serotypes are used for gene therapy (notably AAV1, 2, 5, 6, 8, and 9) and AAV mutants have been generated to optimize long lasting cell transduction without the need for repeated interventions, while evading host immune responses.

### AAV Transduction of Cells

Viral vectors infect target cells by binding to receptors and coreceptors expressed at the cell surface, with ensuing endocytosis in the endosomes. Figure 1 summarizes the different steps of cell transduction by AAV (the general principles of transduction can be applied to other viruses used in gene therapy, with some variations). AAV interact with specific glycans and glycoconjugates present at the cell surface, notably the heparan sulfate proteoglycan.^14^ This interaction is facilitated by various coreceptors, which include fibroblast growth factor receptor 1, hepatocyte growth factor receptor (i.e., c-MET), and a specific AAV receptor.^14^ Subsequently, AAV are released from endosomes following structural changes and enter the nucleus of the cell. In the nucleus, uncoated AAV release their genetic material that usually remain episomal (not integrated into host genome) and is converted into double stranded DNA allowing the transcription and translation of the transgene of interest (Figure 1).

**Figure 1:**
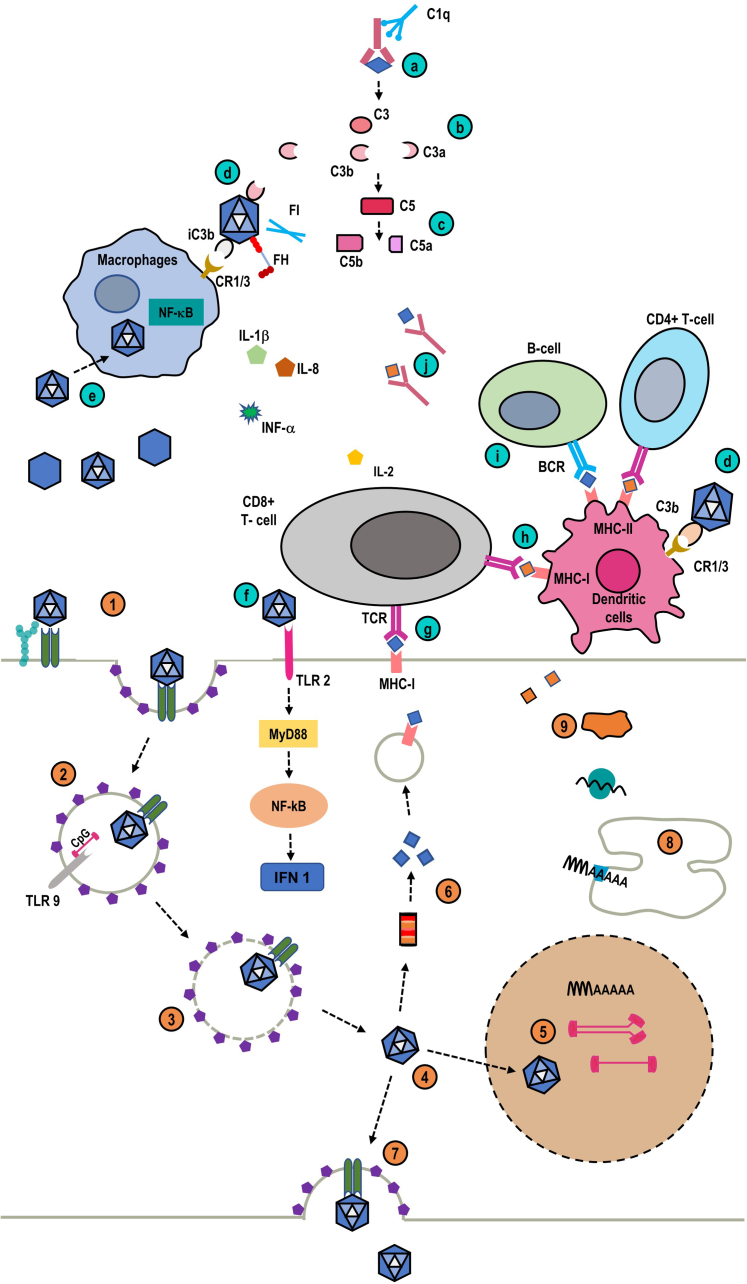
The upper part represents the outside cellular environment and the bottom part represents the interior of the cell. Lower panel. Mechanisms of cell transduction by viral vectors. The mechanisms are shown for adeno-associated virus (AAV), the most widely used viral vectors, but apply to other viral vectors with some variations. (1) AAV attaches to specific glycosylated molecules at the surface of a host cell, and this attachment is probably facilitated by coreceptors and a specific AAV receptor. (2) Subsequently, AAV undergoes internalization through a predominantly (but not exclusively) clathrin-dependent mechanism in endosomes. (3) Low pH in endosomes facilitates a conformational change of major capsid proteins VP1 and VP2, (4) followed by endosomal escape, (5) transport to the nucleus, and uncoating of the genetic material. (6) A part of the viral capsids undergoes degradation in the proteasome, with the release of capsid-derived antigens or (7) is exported via transcytosis. In the nucleus, single-stranded DNA, that are transcriptionally inert, are converted to double-stranded DNA by DNA polymerases prior to transcription. Some AAV? are self-complementary and contain sense and antisense genome that can annihilate in the nucleus. Inverted terminal repeats in the AAV drive intermolecular and intramolecular recombinations to form episomal genomes. The viral vector genome very rarely integrates into the host cell genome. (8) Therapeutic gene transcripts subsequently undergo translation and (9) posttranslational maturation to produce the protein of interest. Upper panel. Host immune responses to viral vectors and produced proteins. (a) One of the host immune responses to the administration of viral vectors (some of which are empty, i.e., devoid of genic material) is the activation of the complement classical pathway through antiviral preformed (or newly formed) neutralizing antibodies, through the binding of C1 complex. (b) Classical pathway activation, that can be amplified by the activation of the alternative pathway, leads to the generation of C3a (an anaphylatoxin) and C3b. (c) Ultimately, complement pathways converge toward the activation of C5 with the release of C5a (an anaphylatoxin) and C5b that mediates complement cytotoxicity via the membrane attack complex. (d) C3b binds to viral capsids and is converted to inactive iC3b through the combined action of factor H (FH) and factor I (FI). iC3b mediates the opsonization of viral capsids and their uptake by macrophages or dendritic cells (via the interaction with the complement receptors 1 and 3 (CR1/3). (e) Viral capsids can also transduce monocytes or macrophages and activate these cells in a NF-kB-dependent mechanism, leading to the release of various inflammatory cytokines (interleukin 1 beta and 8 [IL-1β, IL-8], and interferon-alpha. Conserved molecular motifs, the pathogen-associated molecular patterns, expressed on viral capsids are recognized by pattern recognition receptors, (f) such as Toll-like receptors at the surface of host cells, with subsequent activation of the myeloid differentiation primary response 88 (MyD88) and NF-kB pathway and the synthesis of type 1 interferon. (g) The expression of viral antigens associated with class I major histocompatibility complex expressed on host cell surfaces, followed by the interaction with cognate T-cell receptors generates cytotoxic T cells. Similarly, (h) presentation of viral-derived peptides by dendritic cells to T helper (Th) cells and (i) B cells (via the B-cell receptor) (j) amplifies the immune response with the generation of specific antiviral or antipeptide antibodies.

### Host Immune Responses to Viral Vectors Used in Gene Therapy

Viral vectors are used for gene therapy because they have acquired and refined throughout evolution an ability to transduce human cells. The human immune system has developed in parallel different elaborate immune mechanisms that aim to control viral infections. Thus, viral vectors used in gene therapy may activate host immune responses, especially when infused systemically at high dose (Figure 2). The induced immune response varies depending on the vector serotype and dose, preexisting immunization, the route of administration, and potentially the nature of the transgene.

**Figure 2:**
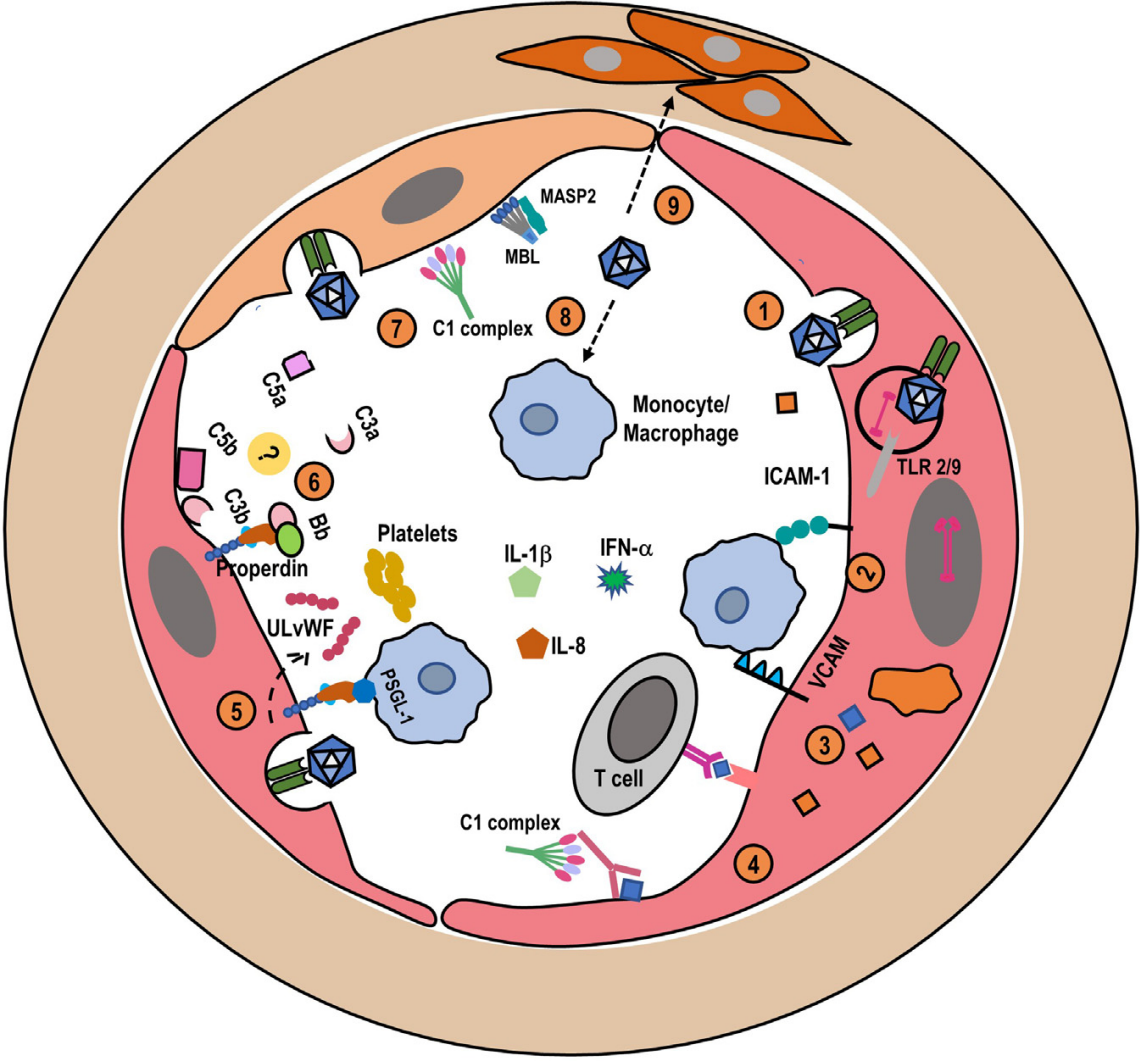
Potential mechanisms of thrombotic microangiopathy associated with viral vector–based gene therapy. Several coexistent mechanisms, some of which are speculative, may contribute to the occurrence of thrombotic microangiopathy in patients receiving viral vector–based gene therapy. (1) Viral vectors may transduce endothelial cells (2) with subsequent activation of these cells and the expression at their surface of the inflammatory molecules intercellular adhesion molecule-1 and vascular cell adhesion molecule. Sensing of the viral genome by Toll-like receptors in endosomes may also contribute to endothelial cells activation. The expression at endothelial cell surface of antigens derived from viral vectors or produced proteins leads to (3) an antiendothelial cell cellular and (4) a humoral reaction. (5) Transduced endothelial cells release increased amounts of ultra large von Willebrand Factor multimers and express properdin that promote platelet-monocyte interactions (via P-selectin glycoprotein Ligand 1), and platelets aggregation. (6) It remains unknown whether properdin acts as a docking platform for the alternative C3 convertase on endothelial cells, and triggers or amplifies complement activation. (7) Transduced activated and apoptotic endothelial cells (in the setting of high viral vectors load) can trigger the activation of the classical and lectin (mannose-binding lectin and mannan-binding lectin serine protease 2), pathways. (8) Direct transduction of monocytes/macrophages by viral capsids releases inflammatory cytokines that contribute to endothelial cells activation. (9) Finally, the role of the transduction of smooth muscle cells in the vessel wall in vascular damage is unknown.

The early step of this immune response involves innate immunity and relies on 2 main mechanisms (Figure 1). The first response against viral vectors is based on the recognition by specialized receptors (pattern recognition receptors, such as Toll-like receptors) of conserved molecular motifs, the pathogen associated molecular patterns, on invading viruses. The second mechanism involves the binding of preformed (or newly formed) antibodies directed against viral vectors antigens with subsequent activation of the complement classical pathway and potentially the lectin pathway. Thus, patients previously immunized against viruses used as therapeutic vectors, are usually not eligible for gene therapy because administration of viral vectors may lead to an overwhelming inflammatory response, whereas neutralizing antibodies will reduce the efficiency of target cells transduction.

Complement is a central part of innate immunity. The classical^15^ (viral capsid inactivation by C4,^15^ C1q-dependent generation of antiviral antibodies^16^) and the alternative pathways (C3-dependent cytokine release, factor B–dependent generation of antiviral antibodies^16^) have been implicated in the innate response to viruses, including adenoviruses. However, to date whether viral vectors directly activate the complement system remains a debated question.^17^ Besides, several viruses have developed escape strategies of innate immunity including complement system, which include the recruitment of inhibitors such as complement factor H or complement factor I, the stabilization of membrane bound complement regulators (CD55, CD46, and CD59) and/or viral gene expression of complement like proteins either directly integrated in viral envelope or protecting host cells through soluble or membrane bound complement regulators.^18^^,^^19^ For instance, AAV, at low concentrations, avoid complement activation through complement factor H binding.^17^^,^^20^^,^^21^ These strategies of complement evasion are even used for viral vectors engineering, and AAV have been designed to integrate a gene coding for membrane bound decay accelerating factor that enhances degradation of complement proteins.^22^

Nevertheless, complement mediates the main part of the toxicity of neutralizing antiviral antibodies. Furthermore, complement activation fragment iC3b contributes to the opsonization of viral vectors and their uptake by monocytes or macrophages. This is followed by the activation of these cells leading to the expression of NF-kB-dependent inflammatory genes (IL1-β, IL-8, macrophage inflammatory protein 2^17^) (Figure 1). Complement activation also promotes the antiviral humoral response, notably through cross-linking of the B cell receptor and complement receptor 2 at the B-cell surface.^23^ Of note, like other microorganisms, AAV can bind the alternative pathway inhibitor, factor H, as a protection against complement induced lysis.^17^ This may explain the relatively reduced ability of AAV to activate complement and the generally mild inflammatory reaction induced by these agents, at least *in vitro*.

The second line of immune response involves adaptive immunity through the following: (i) the generation of specific T and B cells reactive to viral antigens (in naïve patients) or transduced gene products, and (ii) the release of inflammatory cytokines (Figure 1).

Immune response to viral vectors may differ from the 1 occurring during a natural viral infection. Viral vectors are nonreplicative products, delivered at a very high single dose, and potentially introduced at an unnatural site (eye or muscle, for instance). Besides, AAV have been engineered to evade to some extent immune responses through the following: (i) the use of tissue specific promotors in order to restrict AAV transduction to target cells,^10^^,^^24^ (ii) a decreased recognition by Toll-like receptors in endosomes (via depletion of the vector genome of CpG sites, or insertion of Toll-like receptors inhibitory sequences^20^),^25^ and (iii) the evasion from preexisting neutralizing antibodies.

### The Side Effects of an Innovative Therapeutic Tool

Gene therapy is increasingly used in clinical practice for an expanding spectrum of neurological, muscular, cardiovascular, ophthalmological, and hematological disorders. The clinical efficacy of gene therapy has been demonstrated in several studies^26^, ^27^, ^28^, ^29^ but treatment related side effects have also been recognized. The most frequent side effect is elevated liver enzymes that has been linked to viral transfection of hepatocytes in the setting of an important viral vector load (Table 1). Other side effects include systemic reactions, thrombocytopenia and TMA, TMA-like disorders or immune mediated acute respiratory distress syndrome.^40^ Most of these side effects appear to be dose-dependent and tend to occur more frequently in older patients with a higher body surface (viral vector dose being adjusted to body weight).

**Table 1:** Main potential complications of viral vector–based gene therapy

Type of adverse event	Estimated incidence	Mechanisms of adverse event
Increased liver enzymes levels. Hepatitis.	25%–90% of patients may have subclinical elevation of liver enzymes within 7 days of after i.v. infusion of high-dose viral vectors^30^^,^^31^ (toxicity delayed up to 4–10 wk for low-dose viral vectors infusion), with a 2nd peak at 1 mo. May occur after intrathecal administration. Rare cases of fatal acute liver toxicity have been reported.^32^	High frequency probably related to high hepatic tropism of various viral vectors, high liver vascularization with the presence of fenestrated sinusoidal endothelial cells.^33^ Transduction and expression of AAV, leads to direct early activation by viral vectors of Kupffer cells (resident liver macrophages) with TNFα release and of hepatocytes (NF-KB pathway).^34^ Anticapsid T cells and antibodies also contribute to liver toxicity. Liver histology shows hepatocytes hypertrophy with scattered necrosis, and T cell infiltration in severe cases. Not fully prevented by corticosteroids and other immunosuppressive regimens.
Thrombocytopenia. Usually transient, asymptomatic, and viral vector dose-dependent. Occurs within 6–8 d after viral vectors infusion.	Up to 75%–90% of patients.^26^^,^^35^	Direct activation of platelets by some viral vectors (adenovirus).^36^ Increased expression of P-selectin on platelets and endothelial cells, and increased release of ultralarge von Willebrand multimers by endothelial cells. Increased release of microparticles by platelets and endothelial cells. Enhanced leucocytes, platelets, and endothelial cells interaction, through the binding of P-selectin to PSGL-1.^36^
Hemophagocytic lymphohistiocytosis.	Rare reports.	Cytokine release following monocytes viral transduction and B-cell and T- cell activation.^37^^,^^38^
Thrombotic microangiopathy	< 1%–2%	See Figure 2.
Dorsal root ganglion pathology.	< 1%.	Neuronal and axonal direct viral toxicity.^39^

AAV, adeno-associated virus; PGSL, P-selectin glycoprotein Ligand 1.

### Gene Therapy–Associated TMA

TMA has emerged as a rare but severe potential complication of gene therapy. Since 2020, 13 cases of TMA occurring after gene therapy have been reported, exclusively in children as expected due to the indications of therapy. The incidence of TMA is hard to ascertain but is probably lower than 1% to 2%. Reported cases of gene therapy-related TMA are summarized in Table 2. TMA occurred within 8 days following gene therapy, that involved systemic administration of high dose of AAV9 vectors in the vast majority of cases. AAV9 is the most widely used viral vector especially in 1 of the first approved gene therapy, onasemnogene abeparvovec (Zolgensma) used for spinal muscular atrophy. TMA was mostly characterized by profound thrombocytopenia and acute kidney injury of variable severity (1 patient required hemodialysis). One patient had a kidney biopsy at a late stage of the disease, that showed pathological features of TMA. Extrarenal manifestations were noted as follows: elevated pancreatic enzymes (*n* = 1), skin damage (*n* = 1), hepatic dysfunction (*n* = 1), and congestive heart failure (*n* = 1). Four tested patients had at least 1 feature of systemic complement activation and 2 patients underwent complement gene testing that revealed a variant of unknown significance in complement factor I gene. Treatment consisted of plasma exchanges (*n* = 3) and/or eculizumab (*n* = 4), and/or corticosteroids (*n* = 1). TMA resolved in all patients except 1 who ultimately died in the setting of sepsis (dialysis catheter) and recurrence of TMA.

**Table 2:** Reported cases of thrombotic microangiopathy (TMA) occurring in patients undergoing viral vector–based gene therapy

Reference	Number of patients (age)	Type of GT / viral vector (dose) / disease	Time from GT to TMA (d)	Plt count (G/l)	SCr (μmol/l)	Features of complement activation (*n*, %)	Management	Follow-up	Outcome	Special features
Chand *et al.*,^41^ 2020	3 (5 / 12 / 14 mo)	onasemnogene abeparvovec (Zolgensma) / AAV9 (1.1 10^14^ vg/ kg) / SMA	7–8	11–17	62–82, 26	Low C3 (2/3, 66%) Low C4 (3/3, 100%) High Bb, sC5b–9 (2/2, 100%), normal CH50, normal CFH, CFI	PEX (*n* = 1), Cs, Ecu (*n* = 1)	1 y, 4 w, 3 mo	TMA resolution within 4 wk ; persistent HT (*n* = 2)	No complement gene variant detected
Prabhu *et al.*,^42^ 2020	1 (6 mo)	onasemnogene abeparvovec (Zolgensma) / AAV9 (1.1 × 10^14^ vg/kg) / SMA	5	< 30	53	Normal C3 Low C4, normal CH50	PEX (5 cycles)	NA	TMA resolution; persistent HT	Only abstract
Guillou *et al.*,^43^ 2022	1 (6 mo)	onasemnogene abeparvovec (Zolgensma) / AAV9 (1.1 × 10^14^ vg/kg)/ SMA	8	< 3	228	Low C3, normal C4, high sC5b–9, low CH50, Normal CFH, CFI	HD, Ecu	57 d	Death (day 57)	VUS in *complement factor I* gene
Yazaki *et al.*,^44^ 2022	1 (23 mo)	onasemnogene abeparvovec (Zolgensma) / AAV9 (1.1 × 10^14^ vg/kg) / SMA	5	7	107	Normal C3, low C4, low CH50	HD, Ecu, PEX (4 cycles)	1 y	TMA resolution; HT at 1 y	KB done at 49 d: mesangiolysis, endothelial cell swelling, and partial double contours of the GBM.
Mendonca *et al.*,^45^ 2023	1 (15 mo)	onasemnogene abeparvovec (Zolgensma) / AAV9 (1.1 × 10^14^ vg/kg) /SMA	NA	<120	HD	NA	HD, PI	30 d	CKD	
Witte *et al.*,^46^ 2022	1 (4 y)	onasemnogene abeparvovec (Zolgensma) / AAV9 (NA)/ SMA	7	NA	NA	NA	Ecu	NA	TMA remission after 1 week	Only abstract
Poster / Press release										
Shieh *et al.*,^47^ 2021	2 (NA)	SGT-001/ AAV9 (NA) / DMD	NA	NA	NA	NA	NA	NA	TMA resolution	
Belluscio *et al.*,^48^ 2021	2 (NA)	PF-06939926 / AAV9 (NA) DMD	NA	NA	NA	NA	NA	NA	TMA resolution	
Rocket Pharmaceuticals,^49^ 2021	1 (NA)	RP-A501 / AAV9 (1.1 × 10^14^ vg/kg) / Danon disease	NA	NA	NA	NA	HD	NA	TMA resolution	

AAV, adeno-associated virus; CFH, complement factor H; CFI, complement factor I; CH50, complement hemolytic activity 50%; Cs, corticosteroids; DMD, Duchenne muscular dystrophy; Ecu, eculizumab; GT, gene therapy; HD, hemodialysis; HT, hypertension; NA, not available; PEX, plasma exchanges; PI, plasma infusion; Pl, platelet count; SCr, serum creatinine; SMA, spinal muscular atrophy; vg, viral genomes.

### Potential Mechanisms of Gene Therapy-Associated TMA

Potential mechanisms of TMA in the setting of gene therapy are shown in Figure 2. TMA in general arises from 2 main mechanisms. The first is an increased platelet aggregation or activation of the coagulation cascade, as in ADAMTS-13 deficiency-related TMA or thrombotic thrombocytopenic purpura and disseminated intravascular coagulation. The second mechanism involves the direct activation or lesion of the endothelial cells with a transition from a quiescent state to a proinflammatory and prothrombotic state, as in the predominantly renal TMA, the hemolytic uremic syndrome.

Regarding the first mechanism, in experimental models, adenoviral vectors promote the expression of P-selectin on platelets and endothelial cells, increase the release by endothelial cells of ultralarge von Willebrand multimers, leading to the formation of platelet-leucocyte aggregates^36^ (Figure 2). Adenoviral vectors can also directly bind to and activate platelets.^36^ Increased fibrinogen levels and features of coagulation system activation have also been reported in macaques infused with high dose adenoviral vectors.^50^ It is however unknown whether these findings can be translated to AAV.

Direct transduction and ensuing activation of endothelial cells of macrovascular and microvascular beds, by viral vectors (adenovirus and to a lesser extent AAV) have been documented *in vitro* and *in vivo* in a limited number of studies.^51^^,^^52^ This transduction is associated with features of endothelial cell activation, mainly the expression at the cell surface of intercellular adhesion molecule-1 and vascular cell adhesion molecule, leading to the attachment of inflammatory cells and ultimately inflammatory infiltration of the vessels wall (Figure 2). The release of inflammatory cytokines by transduced monocytes or T and B cells may amplify the activation of endothelial cells. Transduction of endothelial cells may also lead to the surface expression of viral peptides and/or of transduced gene product epitopes triggering an endothelial cell–directed T and B cell–specific response.

### Does Complement Play a Role in Gene Therapy-Associated TMA?

Although complement activation occurs after the administration of viral vectors (Figure 3), the role of complement in gene therapy-induced TMA remains ill-defined. *In vitro* assays do not necessarily reflect complement dynamics after viral vector infusion *in vivo*. *In vitro* tests do not usually include a cellular compartment and thus do not consider the cell-bound complement regulators and/or cellular damage. Furthermore, *in vitro*, viral vectors are incubated with plasma-derived complement components for an extended duration whereas the viral capsids are rapidly cleared from the circulation after *in vivo* infusion. In an elegant series of experiments, Tian and colleagues showed that adenoviral vectors activate complement through 2 distinct mechanisms *in vitro* and *in vivo*.^53^ *In vitro*, complement activation appears to be dependent mainly on antiviral capsids antibodies. In contrast, *in vivo*, complement activation occurs independently of antibodies, requires C1q, and is most probably mediated by adenoviral-induced damage to cells, which triggers the activation of classical and nonclassical complement pathways. Furthermore, it remains to be determined whether transduction of endothelial cells by viral vectors leads to the expression at their surface of docking proteins for complement (notably properdin), as reported in other settings of endothelial injury leading to TMA^54^ (Figure 2). The role of constitutional dysregulation of the complement alternative pathway has not been documented, to date. Finally, viral vectors may also transduce smooth muscle cells in the arterial wall,^51^ and the contribution of these cells to the pathogenesis of vascular injury warrants further assessment.

**Figure 3:**
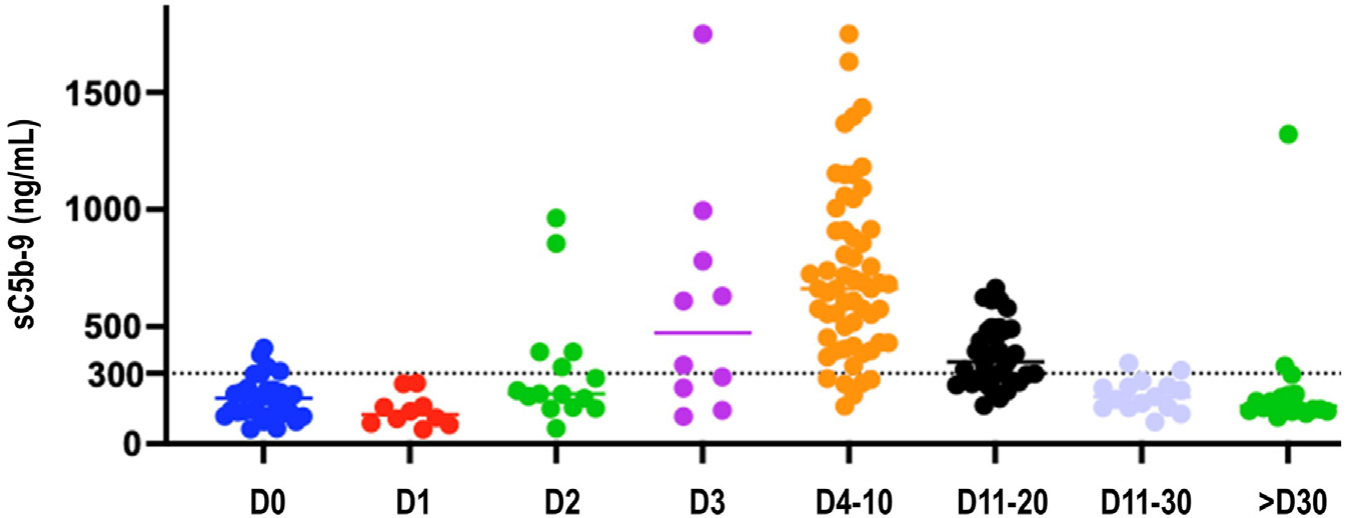
Kinetics of complement final pathway activation in patients receiving viral-based gene therapy. Serum levels of sC5b-9, a marker of activation of the complement terminal pathway, were measured in 54 consecutive patients (28 female; median age 13 months [1–96]) who received zolgensma (AAV9-based gene therapy), for spinal muscular atrophy. D, days after AAV9-based gene therapy infusion (Carine Et Sissy, Isabelle Desguerre and Véronique Frémeaux-Bacchi, personal data). Not all patients had measurements at all time-points. AAV, adeno-associated virus.

Interestingly, complement activation is a hallmark of COVID-19 and the increase in complement activation markers in serum corelates with disease severity.^55^^,^^56^ Furthermore, TMA is a feature of severe COVID-19^57^ and complement mediated atypical hemolytic uremic syndrome triggered by COVID-19 have been reported.^58^

A recent study in patients receiving gene therapy suggests that TMA in this setting is dependent on anticapsid antibodies with the activation of the complement classical pathway and its amplification by the alternative pathway.^59^ The authors argue that the unusual viral presentation to the host immune system (peak concentration of viral vectors within 1 hour of infusion, enormous body area exposed to viral vectors) accelerates the humoral response, with antivirus IgM antibodies detected as early as 5 days after vector infusion, and IgG at 7 days. In this study, all patients without prophylactic immunosuppressive treatment (rituximab and sirolimus) exhibited features of classical and alternative pathway activation, whereas patients with prophylactic immunosuppressive treatment did not. However, none of the patients developed gene therapy–related TMA and only a fraction (6/23) of patients without prophylactic treatment had detectable schistocytes on blood smear. More importantly, complement activation markers peaked at day 5 and preceded the peak of IgM (day 14) and IgG (>21 days).^59^ In the current state of our knowledge, the most probable mechanism of TMA associated with gene therapy seems to be direct endothelial cell injury induced by a high-dose viral load, potentially exacerbated by complement activation as a “second hit.”

### How to Prevent and Treat Gene Therapy-Related TMA?

Even though rare, the potential occurrence of TMA after gene therapy has raised legitimate concerns among patients, families, and health specialists; as well as several practical questions: should patients eligible for gene therapy be screened for complement gene variants as risk factors for TMA? What is the best prophylactic approach in this setting, and should it include anticomplement therapies besides corticosteroids?

In order to answer these concerns and questions, the first step would be to devote more basic and clinical research to dissect the exact mechanisms of gene therapy–associated TMA and the respective contributions of viral vector–induced endothelial cell damage, complement activation (systemic or at the cell surface), and of humoral and cellular immune responses to viral vector infusion. Based on the scarce available data, the benefit of anticomplement therapies is hard to ascertain and is probably less dramatic as in another complement dependent form of TMA, the atypical hemolytic uremic syndrome; in keeping with the assumption that complement activation may be a secondary actor in gene therapy–associated TMA. A prophylactic regimen with rituximab and sirolimus has been recently proposed in patients undergoing gene therapy.^59^ This study included patients receiving various viral vectors at highly variable doses. This promising regimen blunted complement activation and antiviral humoral responses, but whether it prevents TMA remains speculative (no patient in the control group without prophylactic regimen developed TMA).

### Conclusion

Gene therapy has brought tremendous hope for patients withs severe life threatening monogenic diseases. Nevertheless, even though rare, TMA following viral vector–based gene therapy is a matter of concern. Further studies are needed to better define the pathogenesis of this severe complication of gene therapy, and the optimal measures to prevent it. Investigating the respective roles of the humoral response and of complement activation in the occurrence of this type of TMA will be a first step toward improving the care of patients receiving viral gene-based gene therapy.

## Disclosure

ID has received consulting honoraria from Novartis and Pfizer. LS has received consulting honoraria from Novartis. Pfizer, Astellas, and RegenexBio. VF-B has received consulting honoraria from Novartis, Roche, Apellis, Alexion, Astra Zeneca, and Sobi. FF has received consulting honoraria from Novartis, Roche, Apellis, Alexion, Astra Zeneca, BioCryst, Sanofi, and Sobi. All the other authors declared no competing interests.
